# Identification, cloning and expression patterns of the genes related to phosphate solubilization in *Burkholderia multivorans* WS-FJ9 under different soluble phosphate levels

**DOI:** 10.1186/s13568-020-01032-4

**Published:** 2020-06-05

**Authors:** Yu-Qing Liu, Ya-Hui Wang, Wei-Liang Kong, Wan-Hui Liu, Xing-Li Xie, Xiao-Qin Wu

**Affiliations:** Co-Innovation Center for Sustainable Forestry in Southern China, College of Forestry, Nanjing Forestry University, Nanjing, 210037 China

**Keywords:** *Burkholderia multivorans* WS-FJ9, Phosphate solubilizing, Whole genome, Phosphate-related genes, Quantitative real-time PCR

## Abstract

As important plant growth-promoting rhizobacteria, phosphate-solubilizing bacteria (PSB) fix nitrogen, dissolve potassium, promote growth, improve the soil micro-environment, and enhance soil fertility. A high-efficiency PSB strain from the pine tree rhizosphere, *Burkholderia multivorans* WS-FJ9, was screened in our laboratory. In this study, using a Bio Screener fully automatic microbial growth curve meter to determine the growth of the WS-FJ9 strain in phosphate-removing medium, the growth and mineral phosphate solubilization of WS-FJ9 were measured by Mo-Sb colorimetry and organophosphate-solubilization plate assays. Second-generation sequencing technology was used to obtain genomic information and to analyze possible phosphate decomposition genes. The related expression levels of these genes under different soluble phosphate levels were determined by quantitative real-time PCR. The results showed that WS-FJ9 had strong adaptability and capacity for mineral phosphate solubilization at low soluble phosphate levels, which is characterized by its low soluble phosphate induction and high soluble phosphate inhibition. The amount of solubilized mineral phosphate could exceed 140 mg/L. The total length of the WS-FJ9 genome was 7,497,552 bp after splicing, and the GC content was 67.37%. Eight phosphate-related genes were selected to determine their expression patterns at different soluble phosphate levels. Among them, *AP*-*2*, *GspE* and *GspF* were only related to organic phosphate, *HlyB* was only related to inorganic phosphate, and *PhoR*, *PhoA*, *AP*-*1* and *AP*-*3* were related to both. The WS-FJ9 strain utilizes multiple pathways for mineral phosphate solubilization, and the solubilization processes of different phosphate sources are interrelated and independent, indicating that the WS-FJ9 strain can adapt to different phosphate source environments and has good potential for future applications.

## Introduction

Phosphorus (P) is an essential nutrient element for plant growth and development. P participates in most plant metabolic processes and is one of the factors that limits crop yield. It has been reported that 74% of the cultivated land in China lacks phosphate; 95% of the phosphorus in the soil is insoluble, and the phosphorus that can be absorbed and utilized by plants is insufficient to meet plant demands (Chen et al. 2017; Blume et al. 2010). To solve this problem, a large amount of phosphate fertilizer is often used to alleviate the problems caused by phosphorus deficiency in agricultural and forestry production. However, long-term application of phosphate fertilizer not only causes soil hardening, acidification and water pollution but may also harm human health (Chaney 2012). In addition, the raw materials of phosphate fertilizer mainly come from nonrenewable phosphate rock. Therefore, improving the utilization rate of soil insoluble phosphate has become the primary limitation in agricultural and forestry development.

Phosphate-solubilizing bacteria (PSB) in the rhizosphere have attracted increasing attention because of their advantages, such as environmental protection, low cost, and high efficiency (Khan et al. 2007; Owen et al. 2015). Over the years, many studies have been carried out on the characteristics and mechanisms of PSB (Lin et al. 2015). It is generally believed that PSB can dissolve insoluble inorganic phosphates by secretion of small molecule organic acids, proton exchange, and complexation and can degrade organic phosphate by secretion of solubilization enzymes, such as phosphatases and proteases (Qin et al. 2019).

At present, research on the phosphate solubilization genes of PSB is mainly focused on genes related to the solubilization of insoluble inorganic phosphate. For example, Kim et al. (2003) transferred a phosphate solubilization gene (PQQ) to *Escherichia coli* transgenically, which significantly improved the phosphate solubilization efficiency of the *E. coli*. Song et al. (2019) cloned the microbial phosphate solubilization gene *GabY* from the red soil of Guangxi Province and induced its expression in *E. coli*, and the recombinant *E. coli* degraded insoluble inorganic phosphate. Research on organic phosphate solubilization genes is focused on phosphatase genes, mainly including acid phosphatase, alkaline phosphatase and inositol hexaphosphatase genes. Fraga et al. (2001) cloned the acid phosphatase gene *napA* and transferred it to *Burkholderia cepacia* IS-16. The activity of acid phosphatase and the phosphate solubilization activity of this strain were significantly increased in vitro. Due to the wide variety of PSB, there are still relatively few studies on phosphate solubilization pathways and expression patterns of phosphate solubilization genes, which need to be further studied.

The *Burkholderia cepacia* complex (Bcc) is widely distributed in the soil and is an important component of plant growth promoting rhizobacteria (PGPR) that can promote the growth of wheat, rice, poplar and other plants (Nishiyama et al. 2010; Van Trân et al. 2000; Li et al. 2014). PSB can not only promote plant growth by fixing nitrogen, dissolving phosphate and secreting plant hormones (Min et al. 2019) but also produce a variety of antibacterial substances (Chen et al. 2019; Zhang 2018), inhibit soil-borne diseases, and antagonize a variety of plant pathogens (Ren et al. 2006). At the same time, Bcc, as a bioremediation agent, can decompose herbicides and pesticides that are difficult to degrade (Li et al. 2013). To date, there are 17 genotypes of Bcc, and *Burkholderia multivorans* belongs to Bcc genotype II. Some of its strains are human pathogens (Varga et al. 2012), while others have antagonistic effects against some plant pathogens (Sijam and Dikin 2005). At present, the research on this bacterium is mostly focused on its antagonistic substances, but there have been few reports on the molecular mechanisms for phosphate solubilization of this bacterium.

A high-efficiency PSB from the rhizosphere of pine trees, *B. multivorans* WS-FJ9, was screened in our laboratory. Previous studies have shown that WS-FJ9 has good ability to promote plant growth, dissolve phosphate and antagonize a variety of plant pathogenic bacteria (Hou 2012), and preliminary studies have explored its solubilization mechanism of inorganic phosphate by transcriptome analysis (Zeng et al. 2017). However, the ability of this strain to degrade organic phosphate and the expression levels of phosphate solubilization genes under different soluble phosphate levels are not clear. In this study, the growth and mineral phosphate solubilization ability of WS-FJ9 under different soluble phosphate levels were determined to explore its phosphate solubilization characteristics when presented with different phosphate sources. To precisely locate the phosphate solubilization genes and systematically understand the phosphate solubilization pathway of this strain, the second-generation genome of this strain was sequenced to mine genes related to phosphate solubilization. Furthermore, the expression patterns of these genes under different soluble phosphate levels were further analyzed to reveal the mechanism of phosphate solubilization and plant growth promotion of this strain at the molecular level.

## Materials and methods

### Strain and culture conditions

The phosphate-solubilizing bacterium *B. multivorans* WS-FJ9 was isolated from the rhizosphere soil of a 28-year-old slash pine (*Pinus elliotii*) in Guangzhuang Forestry Center, Fujian, China (Hou 2012) and deposited in the Chinese Center for Type Culture Collection (Accession No. CCTCCM2011435). The genomic data were uploaded to NCBI (Accession No. JAAGNW000000000). After WS-FJ9 was activated, a single colony was removed and transferred into LB medium and cultured at 28 °C for 10 h at 200 rpm. Then, 1% of the WS-FJ9 strain seed solution was transferred to a phosphate-solubilizing medium with different exogenous soluble phosphate levels. One hundred-microliter samples were transferred from bottles into a 96-well plate with a liquid pipette, and each sample was repeated 3 times. The 96-well plate with the bacterial solutions was placed in a Bio Screener automatic microbial growth curve instrument for determination of their OD values.

### Phosphate solubilization measurement

Five concentrations of exogenous soluble phosphate (0, 1, 5, 10, and 20 mM) were added to Monkina medium (Yang 2014), the National Botanical Research Institute’s phosphate growth medium (NBRIP) (Han et al. 2019). Ten-microliter aliquots of the bacterial suspensions were pipetted to the center of plates, with each soluble phosphate level repeated in triplicate. The growth and phosphate solubilization of the bacterium were observed after 5 days of incubation at 30 °C. The phosphate solubilization activity was determined by the ratio between the clear zone diameter and the colony diameter. One milliliter of the bacterial suspension was inoculated into the Monkina medium and NBRIP broth medium at each of the soluble phosphate levels in triplicate. Medium without bacterial inoculation served as the control. The supernatants of each of the soluble phosphate treatment and control groups were filtered through 0.22-µm-pore-sized medical millexGP filters (Millipore, USA). The concentrations of soluble phosphate in the filtrates were measured using the ascorbate method (Zhang 2008).

### Sample preparation for genome sequencing

The WS-FJ9 strain was washed 3–4 times with 1*PBS until the supernatant was clear. The samples were quickly frozen in liquid nitrogen and stored at − 80 °C. Three tubes of samples were prepared, each of which was approximately 0.5 g. The samples were sent to a sequencing company (Pasano, Shanghai), and high-quality samples of *B. multivorans* WS-FJ9 total DNA were extracted and sequenced.

### RNA extraction and reverse transcription

A bacterial total RNA extraction kit and reverse transcription kit were used according to the manufacturer’s instructions (Vazyme, Nanjing).

### Quantitative real-time PCR

To understand the phosphate-solubilization mechanism of the WS-FJ9 strain from multiple angles, the phosphate solubilization genes from different phosphate solubilization pathways were selected to detect their relative expression levels by qRT-PCR, including the *PhoR* gene responsible for sensing the two-component system of external phosphate sources, which can sense the concentration of soluble phosphate in the outside world; phosphatase genes *AP*-*1, AP*-*2, AP*-*3*, which encode the acid phosphatase gene and are important enzymes regulating phosphorus metabolism; organic acid genes, such as *PhoA*, which encode alkaline phosphatase, which can mineralize the activity of organic acids; and *HlyB*, *GspE*, and *GspF*, which are related to the secretion system responsible for secreting organic acids and enzymes into the environment. Primer 5.0 software was used to design specific primers for quantitative real-time PCR. The specific primers were designed as follows:

The ChamQ ™ SYBR^®^ qPCR Master Mix (Low ROX Premixed) kit and a 7500 real-time instrument (Applied Biosystems, Foster City, CA, USA) were used for qRT-PCR. The kit instructions were followed, and the reaction mixture was prepared on ice (Table 1).

**Table 1:** Specific primers for qRT-PCR of *Burkholderia multivorans* WS-FJ9 phosphate-solubilizing genes

Gene name	Primer (5′-3′)
*PhoA*	F:ATGTCGACTATCAAGCGCAT
	R:CTCACCCACCTTGTAGATGC
*PhoR*	F:ATCCCGATTTCGTCCGCTACCT
	R:CGTTCGAGTTCCGTGATGTCCTG
*AP*-*1*	F:GAAGAAAACCTGGATCCGCG
	R:GGAAGGCGCCGTACAGGTT
*AP*-*2*	F:GGTGCGCAACATCGTGGTG
	R:CCAGACCTTCGGCAGGGTG
*AP*-*3*	F:CGCCTCGTCTGTGGATCTC
	R:GAAGGCGATCTTGGTCAGC
*HlyB*	F:ATGTATTTCGGCACGACGCT
	R:AGGAACGAGGTGGTGAGGGT
*GspE*	F:AACAGGCCTCGGACATCCA
	R:GTCGAGTTGCGCCATGATTT
*GspF*	F:ATCGTGCTGGCGTTCACCTAT
	R:ACCAGTGCCGCACGAAATC

### Statistical analyses

Statistical analyses were carried out using Excel 2010 (Microsoft Corporation, Redmond, WA, USA) and SPSS software (ver. 23.0 IBM Corp., Armonk, NY, USA). Comparisons among treatments were analyzed for significance using Duncan’s new multiple range test.

## Results

### Growth of *Burkholderia multivorans* WS-FJ9 in phosphate-solubilizing medium with different exogenous soluble phosphate concentrations

**Fig. 1:**
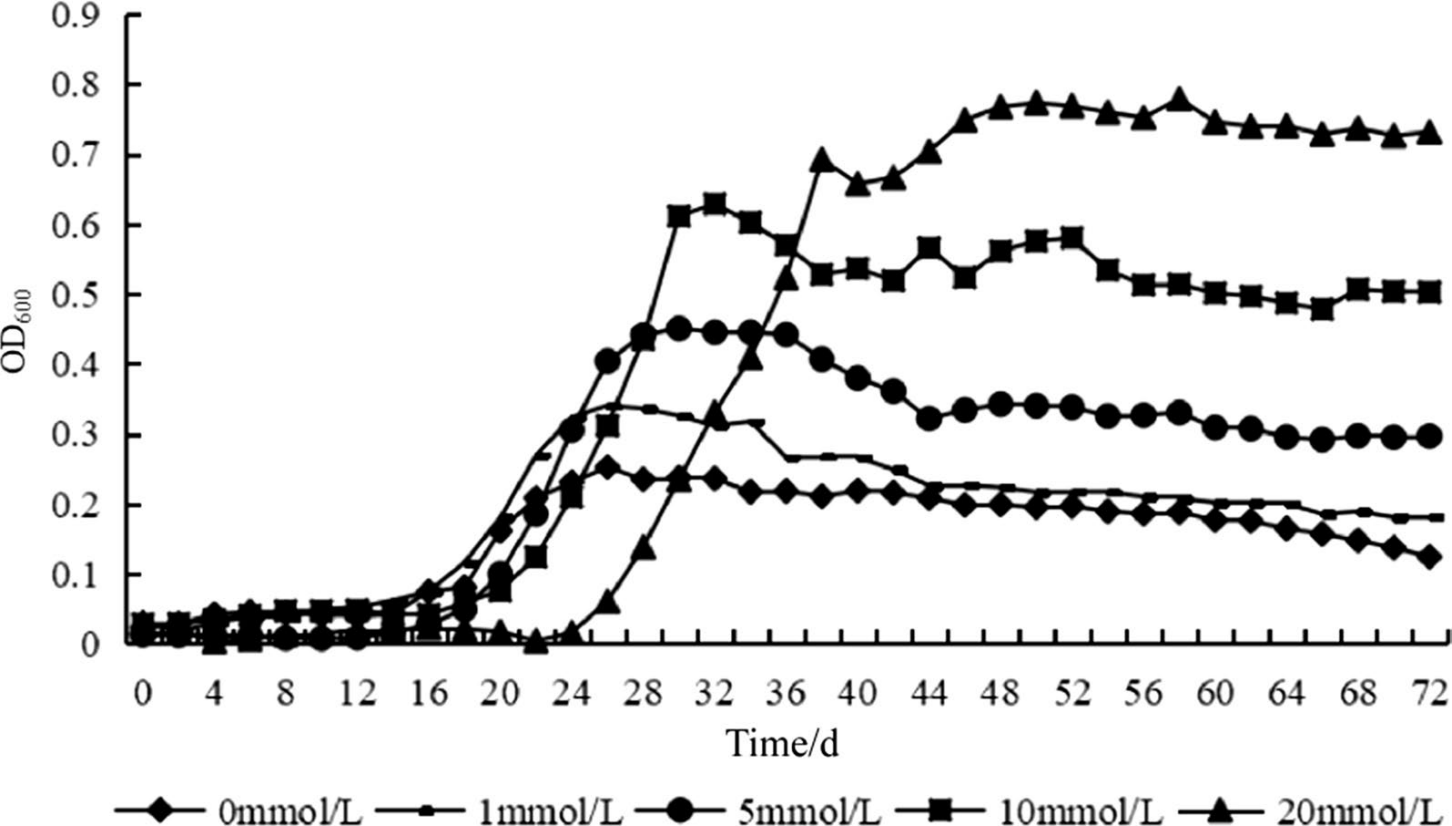
Growth of *Burkholderia multivorans* WS-FJ9 in phosphate-solubilizing medium with different exogenous **s**oluble phosphate concentrations

The growth of *B. multivorans* WS-FJ9 in phosphate-solubilizing medium with different concentrations of exogenous soluble phosphate was examined to better understand the phosphate-solubilizing ability of WS-FJ9 and to determine its phosphate solubilization characteristics. The WS-FJ9 reached the logarithmic phase preferentially under low soluble phosphate conditions in the following sequence: 0 mmol/L ≈ 1 mmol/L > 5 mmol/L > 10 mmol/L > 20 mmol/L (Fig. 1). It is possible that high soluble phosphate concentrations may hinder the early growth of WS-FJ9. However, whether or not the soluble phosphate source is sufficient restricts the total number of viable bacteria in the later stage. The number of colonies in the later stage of logarithmic growth was directly proportional to the soluble phosphate content, that is, 20 mmol/L > 10 mmol/L > 5 mmol/L > 1 mmol/L > 0 mmol/L.

### Detection of phosphate-solubilizing capacity of *Burkholderia multivorans* WS-FJ9

**Fig. 2:**
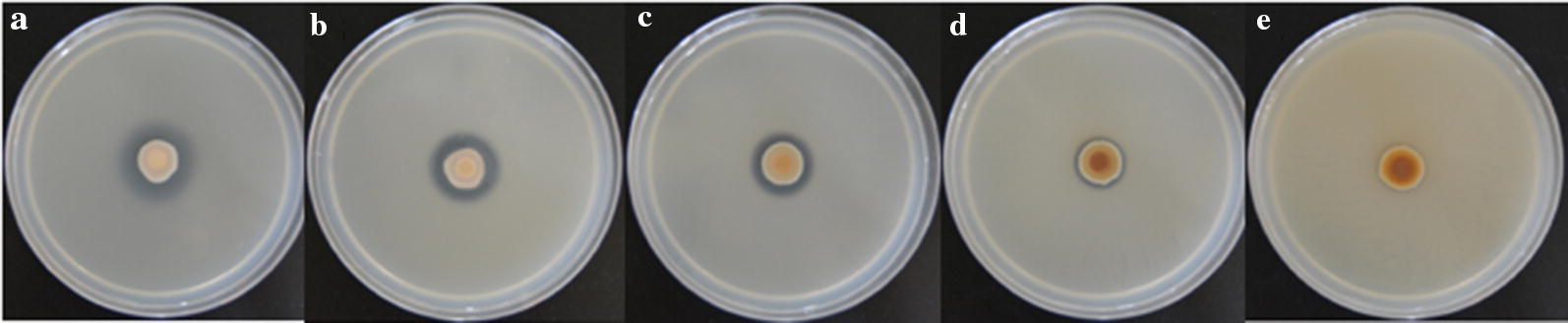
Growth and phosphate solubilization of WS-FJ9 on phosphate-solubilizing plates with different exogenous soluble phosphate concentrations. **a**. 0 mmol/L; **b** 1 mmol/L; **c** 5 mmol/L; **d** 10 mmol/L; **e** 20 mmol/L

The phosphate solubilization by strain WS-FJ9 on a phosphate-solubilizing plate is shown in Fig. 2: with the increase in the soluble phosphate concentration, the diameter of WS-FJ9 colonies also increased, while the diameter of the transparent area decreased. At the same time, the growth of strain WS-FJ9 was consistent with the growth in Fig. 1, which indicated that the growth rate of strain WS-FJ9 (that is, the speed at which the logarithmic growth phase was reached) was not faster with a higher soluble phosphate content in the medium. However, with the increase in soluble phosphate content in the medium, the longer the time that was required to reach the logarithmic growth phase. The final total number of bacteria in each medium depended on the content of soluble phosphate, that is, the higher the content of soluble phosphate, the higher the total number of bacteria in the final medium.

**Fig. 3:**
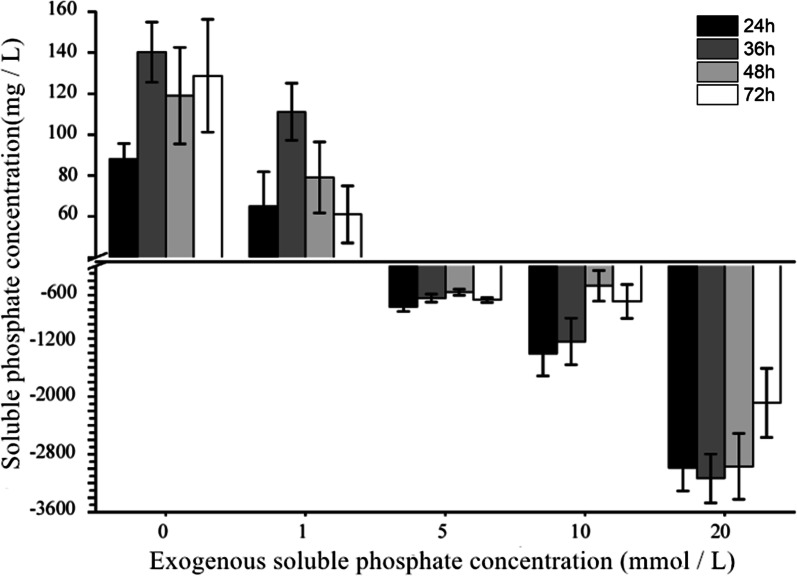
Detection of the phosphate-solubilizing ability of *Burkholderia multivorans* WS-FJ9 under different soluble phosphate levels

According to the phosphate-solubilizing ability of strain WS-FJ9 under different soluble phosphate levels (Fig. 3), the strain showed low soluble phosphate induction and high soluble phosphate inhibition. Under each treatment, the content of soluble phosphate was the highest when the bacteria reached the stable stage and decreased slightly in the later stage. Under the condition of low phosphate, the bacteria reached the stable period the soonest and had the strongest ability to solubilize phosphate. The reason for the slight decrease in the later period may be that the soluble phosphate content in the fermentation broth exceeded a certain threshold value, which inhibited the phosphate solubilization activity of the WS-FJ9.

### Genome assembly and annotation of *Burkholderia multivorans* WS-FJ9

To explore its ability to solubilize phosphate, the genome of strain WS-FJ9 was sequenced. The original reads of the WS-FJ9 strain obtained by sequencing were subjected to quality control, quality evaluation and assembly. The assembled genome characteristics and genome structural prediction are shown in Tables 2 and 3, respectively. The total length of the assembled genome was 7,497,552 bp, and the GC content was 67.37%. A total of 1519 genomic short fragments (contigs) and 479 long fragments (scaffolds) were obtained. The maximum scaffold sequence length was 52,157 bp, the minimum scaffold sequence length was 230 bp, the N50 size was 29,847 bp, and the N90 size was 7350. The genome of strain WS-FJ9 was predicted to encode 7,720 genes covering 5,815,848 bp, accounting for 77.57% of the genome, and the average length of the coding genes was 753.35 bp. In addition, a total of 52 tRNA structures, 85 ncRNA structures, 3 rRNA structures, and 627 CRISPR structures were predicted.

**Table 2:** Statistics of the genomic characteristics of *Burkholderia multivorans* WS-FJ9

Name	Numerical value	Name	Numerical value
Genomic size/bp	7,497,552	Max Scaffold Size/bp	175,577
GC content/%	67.37	Min Scaffold Size/bp	1006
Scaffold quantity	479	Total Scaffold Size/bp	7,497,552
N50	29,847	N90	7,350

**Table 3:** Prediction of the genome structure of *Burkholderia multivorans* WS-FJ9

Name	Numerical value	Total length (bp)
CDS	1519	7,497,552
tRNA	52	4082
rRNA	3	4514
CRISPR	627	2129
ncRNA	85	11,392

### Functional annotation of protein-encoding genes of *Burkholderia multivorans* WS-FJ9

The functional annotation of protein-encoding genes is at the core content of whole-genome analysis of microorganisms and can reveal the biological activities of a species at the molecular level. According to the functional annotation results of the genomic protein-coding genes of WS-FJ9 (Table 4), 6271 protein-encoding genes were compared in the NR database, and 106 protein-encoding genes were compared in the KEGG database. The differences were mainly related to the volume and focus of the databases.

**Table 4:** Functions of the protein-coding genes of *Burkholderia multivorans* WS-FJ9

Annotation in database	No. genes	Percentage of total/ %
NR	6271	81.23
eggNOG	5399	69.94
KEGG	106	1.37
Swiss-Prot	4175	54.08
GO	4545	58.87

### Phosphate-related genes and metabolic pathways of *Burkholderia multivorans* WS-FJ9

The genomic data of strain WS-FJ9 revealed many types of phosphate-related genes, mainly including genes involved in organic acid synthesis and secretion, phosphatase synthesis and secretion and the sensing of external phosphate sources, related to the regulatory system (Table 5).

**Table 5:** Phosphate-related genes of *Burkholderia multivorans* WS-FJ9 (part)

ORF name	Gene name	Function	KEGG
contig122	PhoA	OmpR family	K01077
contig21	PhoB	OmpR family	K07657
contig21	PhoP	OmpR family	K07658
contig448	PhoR	OmpR family	K07636
contig236	AP-1	acid_phos_Burk	K00873
contig55	AP-2	acid_phos_Burk	K00873
contig615	AP-3	A2PA-like-superfamily	K01647
contig1379	HlyB	ABC transpor	K11004
contig22	TolC	Outer membrane protein	K12340
contig21	HlyD	Membrane fusion protein	K11003
contig927	GspD	Secretin	K02453
contig927	GspE	ATPase	K02454
contig927	GspF	IMP	K02455
contig927	GspG	IMP	K02456
contig927	GspH	IMP	K02457
contig927	GspI	IMP	K02458
contig927	GspJ	IMP	K02459
contig927	GspK	IMP	K02460

According to KEGG analysis of this strain, a total of 106 genes of the WS-FJ9 genome were annotated and were enriched in 32 metabolic pathways and could be divided into 9 types. Among them, the pathways with the most genes were mainly signaling and cellular processes (29), genetic information processing (24), metabolism (21), carbohydrate metabolism (18), and metabolism of cofactors and vitamins (13).

**Fig. 4:**
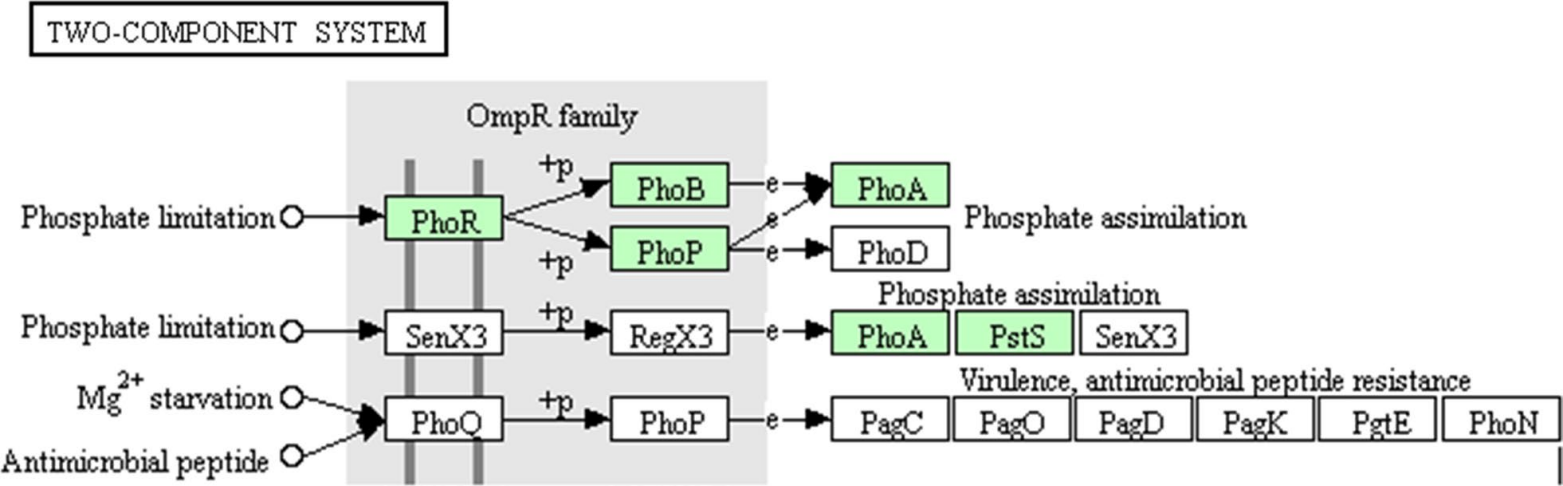
KEGG pathway of genes *PhoR* and *PhoA* of *Burkholderia multivorans* WS-FJ9 (Two-component system)

**Fig. 5:**
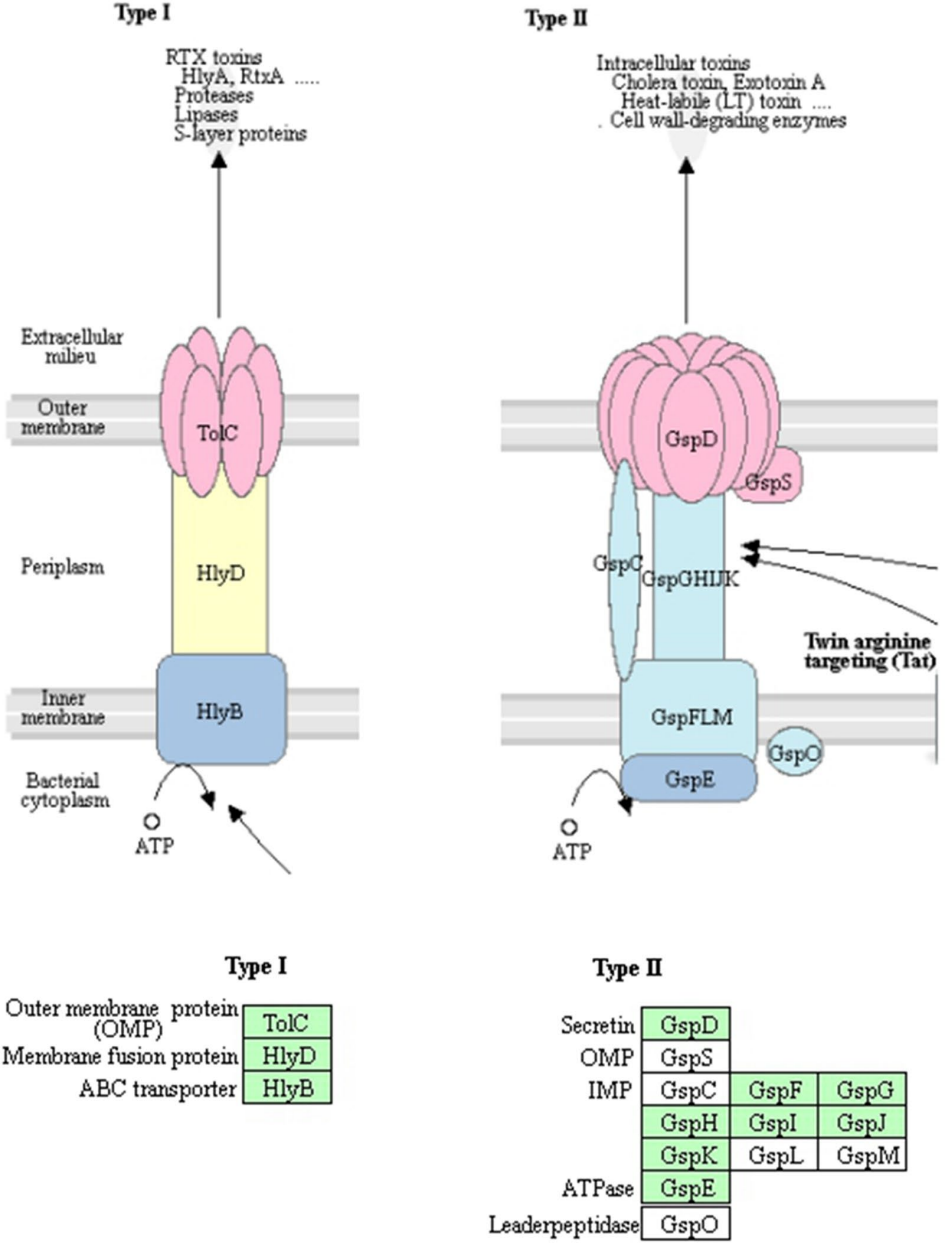
KEGG pathway of the genes *HlyB*, *GspE*, *GspF* of *Burkholderia multivorans* WS-FJ9 (Bacterial secretion system (T1SS, T2SS))

There were more than ten pathways related to phosphate metabolism, including the two-component system (Fig. 4), the bacterial secretion system (Fig. 5), phosphonate and phosphinate metabolism, inositol phosphate metabolism, pentose phosphate pathway, glycerol phospholipid metabolism, oxidative phosphorylation, ABC transport system, phosphotransferase system (PTS), phosphatidylinositol signal system, and the phospholipase D signal pathway. Therefore, the WS-FJ9 strain has the same traditional phosphate metabolism pathways as most of the PSB and has additional phosphate solubilization pathways.

### Quantitative real-time PCR of phosphorus-related genes in *Burkholderia multivorans* WS-FJ9 strains under different exogenous soluble phosphate conditions

The relative expression levels of eight phosphate solubilization genes from different phosphate solubilization pathways were detected by qRT-PCR. The results showed that the expression patterns of the eight genes could be roughly divided into 4 categories:

1. *PhoR* was sensitive to the soluble phosphate concentration and had the same expression patterns in both organic and inorganic phosphate media (Fig. 6b. The gene’s expression level was high under low soluble phosphate conditions (approximately 1.0 times) but was low after the addition of exogenous soluble phosphate, and there was no significant difference in expression (approximately 0.2 mol/0.4 times).

**Fig. 6:**
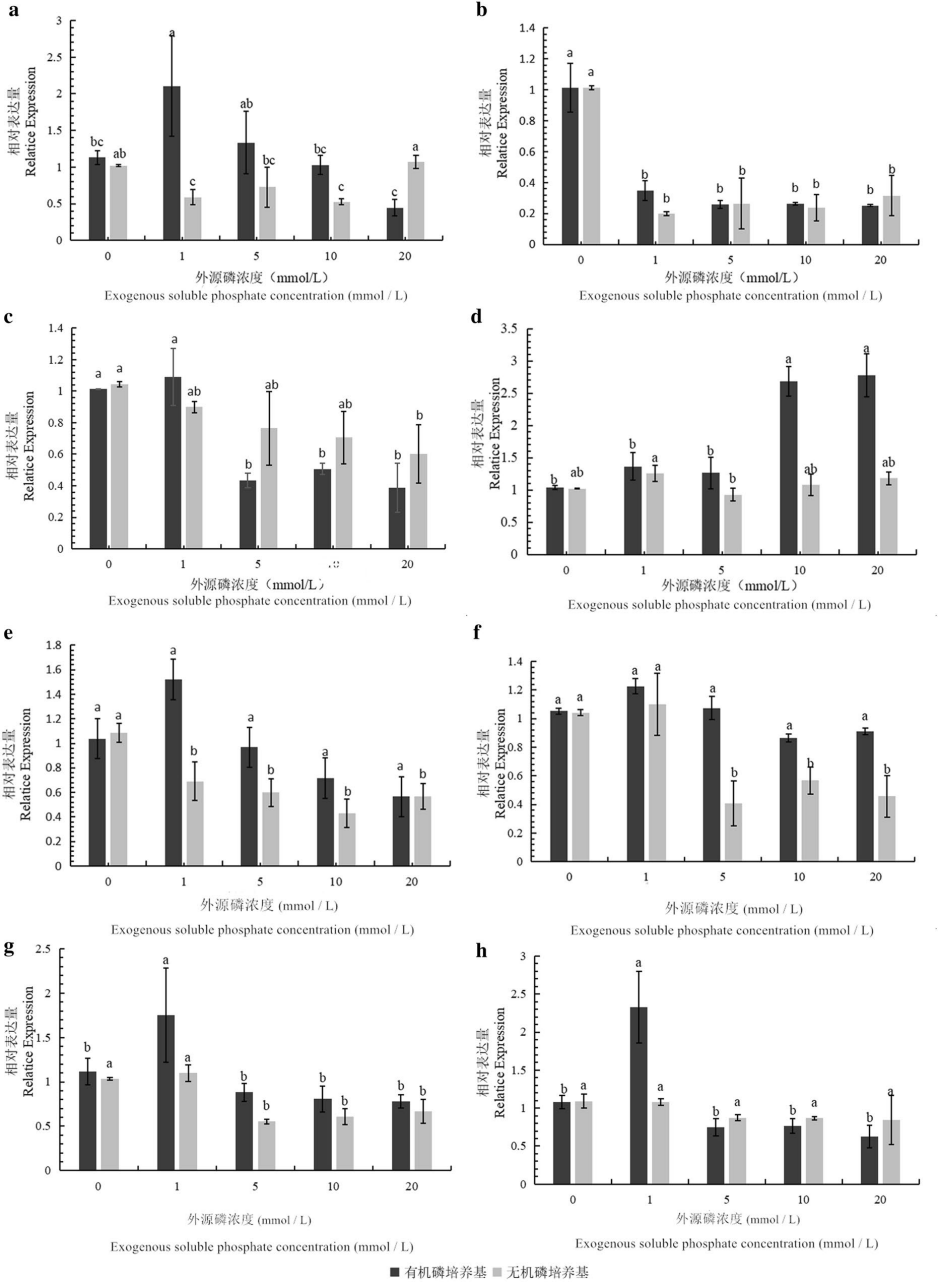
Expression levels of phosphate solubilization genes in *Burkholderia multivorans* WS-FJ9 under different soluble phosphate levels. **a** gene *PhoA*; **b** gene *PhoR*; **c** gene *AP*-*1*; **d** gene *AP*-*2*; **e** gene *AP*-*3*; **f** gene *HlyB*; **g** gene *GspE*; **h** gene *GspF*
2. *PhoA* (Fig. 6a)*, AP*-*1* (Fig. 6c), and *AP*-*3* (Fig. 6e) were sensitive to the soluble phosphate concentration but had different expression patterns in both organic and inorganic phosphate media. In organic phosphate medium with a low exogenous soluble phosphate concentration (0–1 mmol/L), the three genes were upregulated 2.2 times, 1.1 times, and 1.5 times, respectively. When the concentration of exogenous soluble phosphate was high, these genes were downregulated 0.4 times, 0.5 times, and 0.5 times, respectively. In inorganic phosphate medium, the expression level of *PhoA* was slightly higher than in the absence of soluble phosphate and under high soluble phosphate and slightly lower at other levels; the difference, at approximately 0.5 times, was not significant. With the increase in exogenous soluble phosphate concentration, the expression levels of *AP*-*1* and *AP*-*3* decreased, but the differences were not significant. The above three genes had slightly larger responses to the exogenous soluble phosphate content in the organic phosphate medium.
3. *AP*-*2* (Fig. 6d), *GspE* (Fig. 6g), and *GspF* (Fig. 6h) were sensitive to the soluble phosphate concentration only in organic phosphate medium. There was no difference in the expression levels of these genes in the inorganic phosphate medium (all approximately 1.0 times). In organic phosphate medium, *AP*-*2* had a low expression (1–1.3 times) when the exogenous soluble phosphate content was low (0–5 mmol/L) and a high expression (2.7 times) when the exogenous soluble phosphate content was high. The expression patterns of *GspE* and *GspF* were as follows: when the exogenous soluble phosphate concentration was low (0–1 mmol/L), the gene expression level was high, up to 1.7 times and 2.4 times, respectively; when the exogenous soluble phosphate concentration was high, the gene expression level was low, approximately 0.8 and 0.5 times, respectively.
4. *HlyB* was only sensitive to the soluble phosphate concentration in inorganic phosphate medium (Fig. 6f). In organic phosphate medium, that gene showed no differences in expression under different soluble phosphate concentrations (all approximately 1.0). In inorganic phosphate medium, when the exogenous soluble phosphate concentration was low (0–1 mmol/L), the gene expression was upregulated (1–1.3 times). When the concentration of exogenous soluble phosphate was high, the gene expression was downregulated (approximately 0.4–0.6 times).

## Discussion

PSB, as a type of PGPR, can convert insoluble phosphate into available phosphate that can be absorbed and used by crops. At the same time, PSB can fix nitrogen, dissolve potassium, promote plant growth and improve the soil micro-environment. These advantages have attracted widespread attention, and PSB have gradually become a sustainable alternative to solve soil phosphorus deficiency worldwide. *B. multivorans* WS-FJ9 is a high-efficiency PSB obtained from the pine rhizosphere and was examined in our laboratory. This strain can degrade both insoluble organic phosphate and inorganic phosphate. The amount of phosphate solubilized by the WS-FJ9 strain under different soluble phosphate levels reached approximately 140 mg/L. At the same time, WS-FJ9 can dissolve inorganic phosphate at up to 6.2 mM (approximately 1860 mg/L) (Zeng et al. 2017). Guo et al. (2018) screened four strains of PSB from rhizosphere soil of jujube. Among the strains, *Bacillus* sp. P7 and *Acinetobacter* sp. P13 had the strongest ability to decompose organic phosphate, and the amount of solubilized phosphate reached 118.84–127.74 mg/L. Jin et al. (2016) screened and isolated an organophosphate-dissolving bacteria *Stenotrophomonas maltophilia* JYD-4 from *Taxus chinensis* var. *mairei* rhizosphere, and the amount of solubilized phosphate reached 72.38 mg/L. Teng et al. (2019) isolated and characterized 11 kinds of phosphate-solubilizing bacteria from rhizosphere soils of the Yeyahu Wetland, and *Pseudomonas* sp. J-IP1 had the strongest ability to resolve inorganic phosphate, up to 430.40 mg/L. Ibarra-Galeana et al. (2017) isolated 3 kinds of phosphate-solubilizing bacteria from maize rhizospheric soils of northern Sinaloa, of which *Sinorhizobium meliloti* had the strongest phosphate-dissolving ability of approximately 592.85 mg/L. The WS-FJ9 strain has the ability to degrade organic phosphate and inorganic phosphate, and the comprehensive phosphate-dissolving ability belongs to the upper and middle levels. Therefore, the WS-FJ9 strain has a wide application range and potential on phosphorus-deficient soil.

To explore the phosphate solubilization mechanism of the strain, the whole-genome shotgun strategy was used along with second-generation sequencing technology (next-generation sequencing, NGS). The total length of the assembled genome was 7,497,552 bp, and the GC content was 67.37%, which is relatively high, indicating that the gene density is relatively high and that the ability of this strain to resist high temperatures and an alkaline environment is also strong (Zhou 2014). Through KEGG pathway analysis, multiple pathways related to dephosphorization were found in this strain, including a two-component system, bacterial secretion system, phosphonate and phosphinate metabolism, inositol phosphate metabolism, pentose phosphate pathway, glycerol phospholipid metabolism, oxidative phosphorylation, ABC transport system, phosphotransferase system (PTS), phosphatidylinositol signal system, and the phospholipase D signal pathway. The existence of a two-component system and a bacterial secretion system strongly explained the phosphate-solubilizing abilities of the strains that exhibited “low soluble phosphate induction and high soluble phosphate inhibition” in previous research, as well as the secretion of organic acids and phosphatase to degrade insoluble inorganic and organic phosphate.

Previous studies on the phosphate solubilization pathways of PSB mainly focused on the analysis and discussion of the pathways related to organic acid and phosphatase synthesis (Yin et al. 2011). Studies have shown that the phosphate-solubilizing ability of PSB is mostly regulated by the concentration of exogenous soluble phosphate, and most of the organic acids, enzymes and other substances involved in phosphate solubilization are secreted externally to degrade insoluble phosphate (Zeng et al. 2017; Geng et al. 2019). On the basis of the above, this study has increased our understanding of two phosphate solubilization systems that “communicate” between the strain and the outside environment, namely, the two-component system and the bacterial secretion system. Alexander found that there were significant differences in the expression levels of the *PhoA*, *PhoC*, and *PhoD* genes controlled by phosphate in *Streptomyces coelicolor*. Under low soluble phosphate conditions, *PhoA* and *PhoD* were upregulated, while *PhoC* showed the opposite expression patterns (Apel et al. 2007). In this study, the expression of *PhoA* in the WS-FJ9 strain was consistent with that in *S. coelicolor*, and both showed high expression under low soluble phosphate. However, the expression patterns of genes related to the bacterial secretion system under different exogenous soluble phosphate conditions have not been reported; therefore, this study selected key genes in the type I and type II secretion systems for analysis. By measuring the expression profiles of phosphate solubilization genes in strain WS-FJ9 at different soluble phosphate levels, we found that genes from different phosphate solubilization systems and pathways have different expression patterns. *AP*-2, *GspE*, and *GspF* were sensitive to the soluble phosphate concentration only in organic phosphate media, and *HlyB* was sensitive only in inorganic phosphate medium. *PhoR* was sensitive in both organic and inorganic phosphate media. *PhoA, AP*-*1,* and *AP*-*3* were sensitive to the soluble phosphate concentration, but they had different expression patterns in both organic and inorganic phosphate media. These findings indicate that these genes are directly or indirectly regulated by soluble phosphate and play an important role in responding to exogenous soluble phosphate.

Because the phosphate-solubilizing characteristics of WS-FJ9 were induced by low-soluble phosphate and high-soluble phosphate inhibition, that is, regulated by the soluble phosphate concentration, it is speculated that the genes that are sensitive to the soluble phosphate concentration and show high expression play an important role under this condition. Yang et al. (2016) found that the phosphate solubilization genes *GDH* of *Pseudomonas* sp. Wj1 and *Enterobacter* sp. Wj3 have different expression patterns under different soluble phosphate levels. It is speculated that the two strains have different phosphate solubilization mechanisms. Therefore, and in view of the multiple expression patterns of the phosphate-solubilizing genes of strain WS-FJ9, we speculate that this strain has multiple phosphate solubilization mechanisms, which are regulated by phosphate solubilization genes from different phosphate solubilization pathways. These genes are not only related to and independent of each other in the solubilization of organic phosphate and inorganic phosphate but also support each other, which enables strain WS-FJ9 to adapt to different phosphate source environments. Therefore, the strain has good application prospects. In the future, we can examine phosphate-sensing genes or secretion system genes to gain a more comprehensive and in-depth understanding of the phosphate-solubilizing mechanism of this strain. In addition, the genomic data obtained from strain WS-FJ9 can be used to accurately locate relevant genes, which provides a good basis for future research on energy and material metabolism pathways and the related regulatory mechanisms.

## Data Availability

All the data and materials have been provided in the main manuscript.
